# Supplementary figures and images for: Regularized estimation of large-scale gene association networks using graphical Gaussian models
Source: BMC Bioinformatics. 2009 Nov 24;10:384. doi: 10.1186/1471-2105-10-384 (PMC2808166; doi:10.1186/1471-2105-10-384)

**3 clusters**

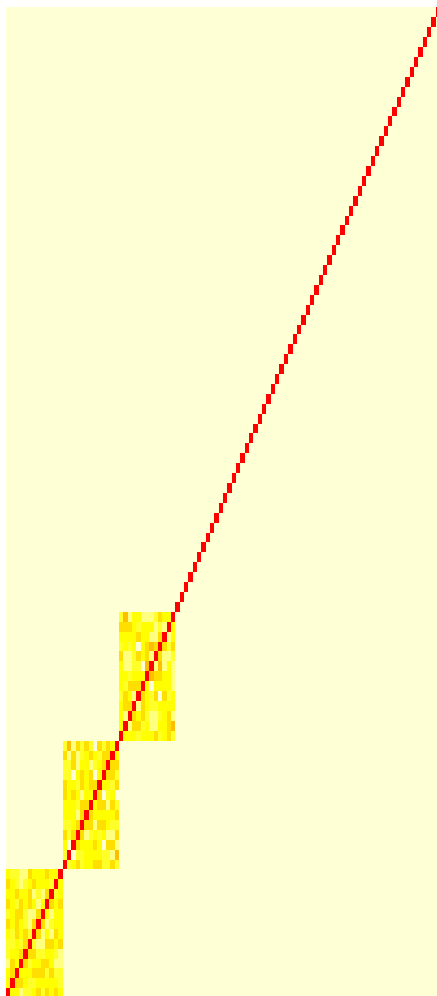

**3 stars**

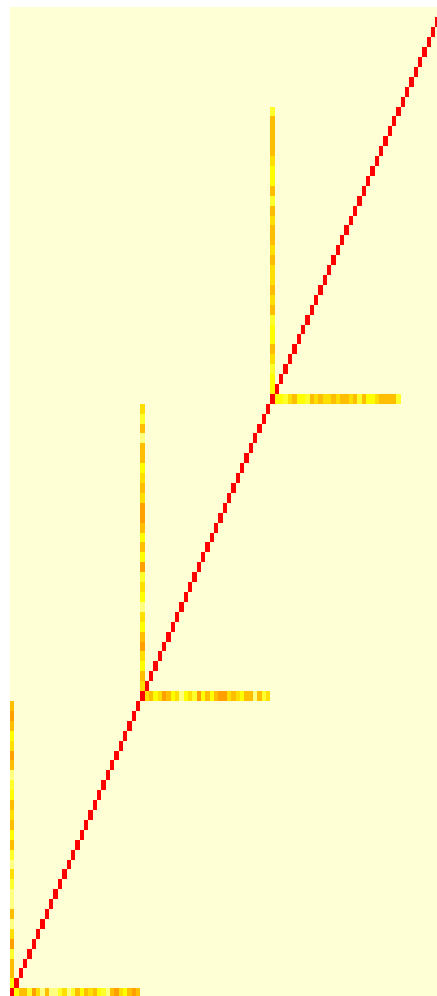

Supplement: Additional file 1 — Cluster structure. The figure illustrates the two different cluster structure (denoted by "clusters" and "stars") that is used in the simulation study. [file 1471-2105-10-384-S1.PDF]

**0.05**

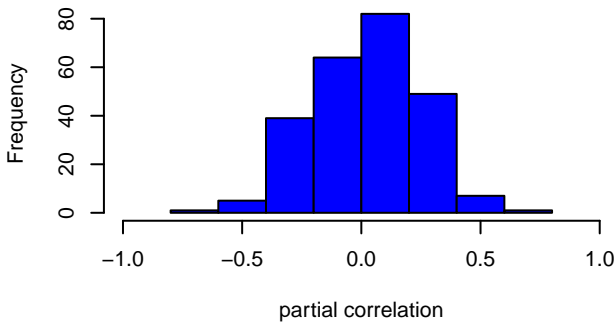

**0.1**

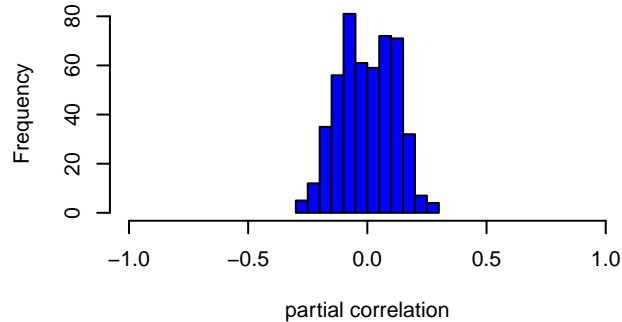

**0.15**

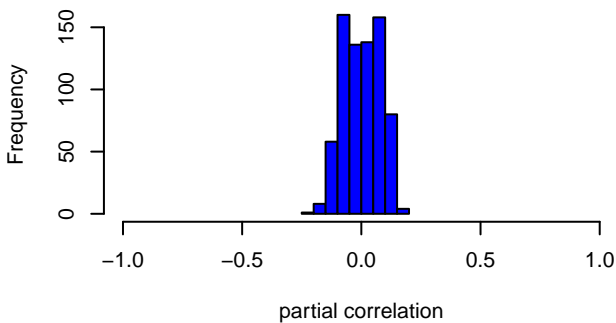

**0.2**

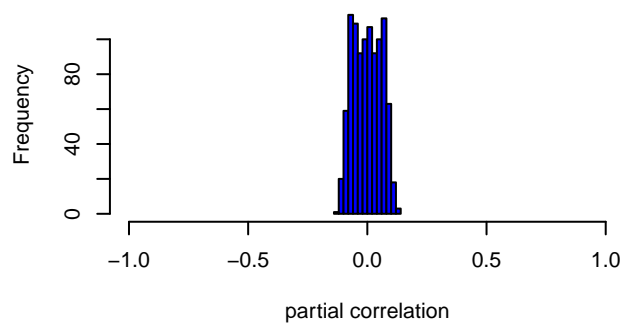

**0.25**

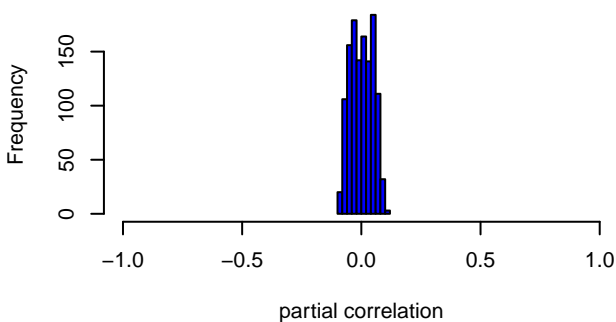

Supplement: Additional file 2 — Histogram of partial correlations. The figure displays the histogram of the non-zero partial correlations in the simulation study for different density levels. [file 1471-2105-10-384-S2.PDF]

**n= 25**

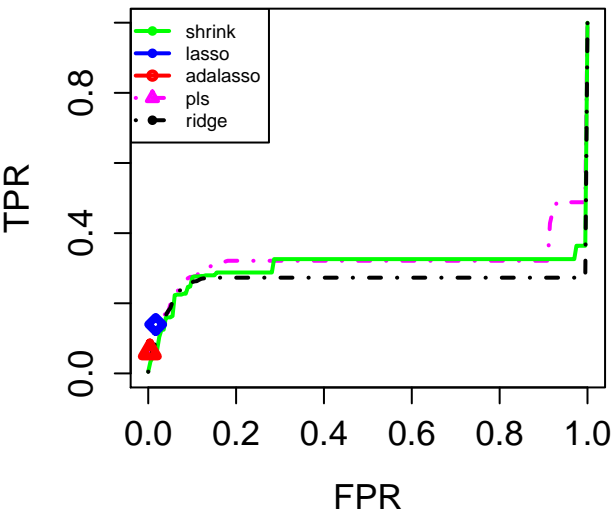

**n= 50**

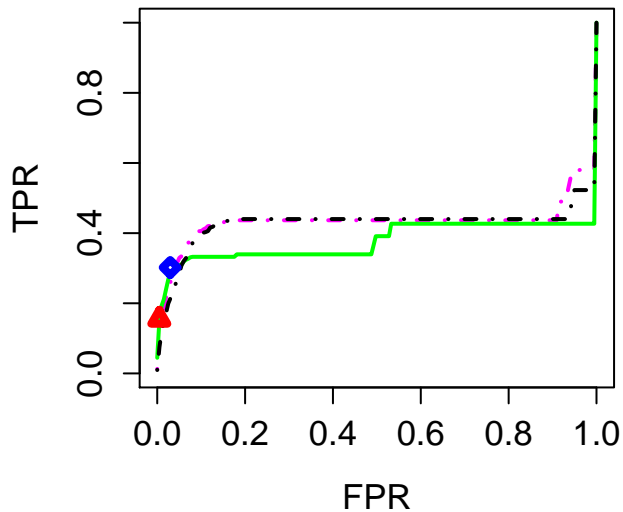

**n= 75**

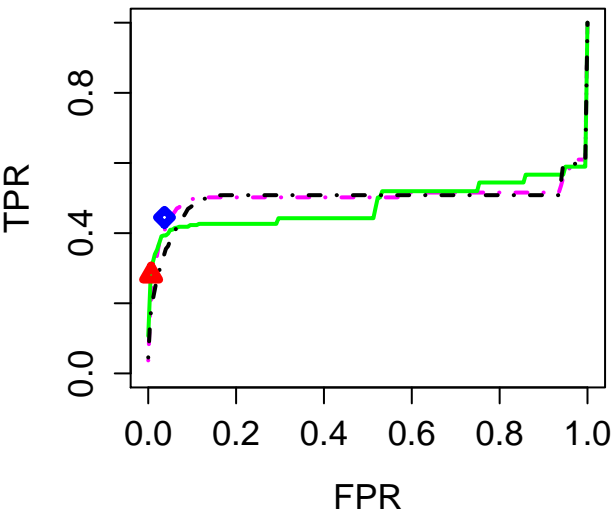

**n= 100**

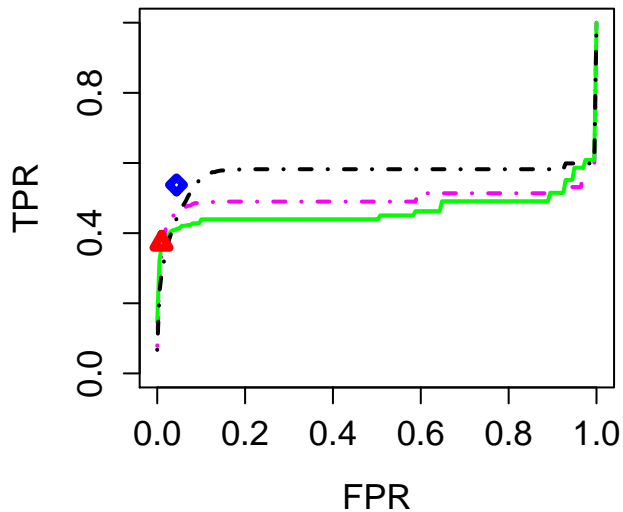

Supplement: Additional file 3 — ROC-curves for a density of 5%, part I. The figures display the ROC-curves for a density of 5%, and for n = 25; 50; 75; 100 [file 1471-2105-10-384-S3.PDF]

**n= 25**

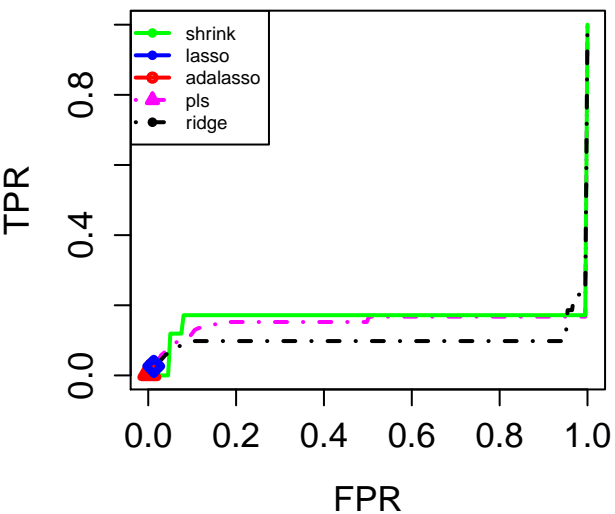

**n= 50**

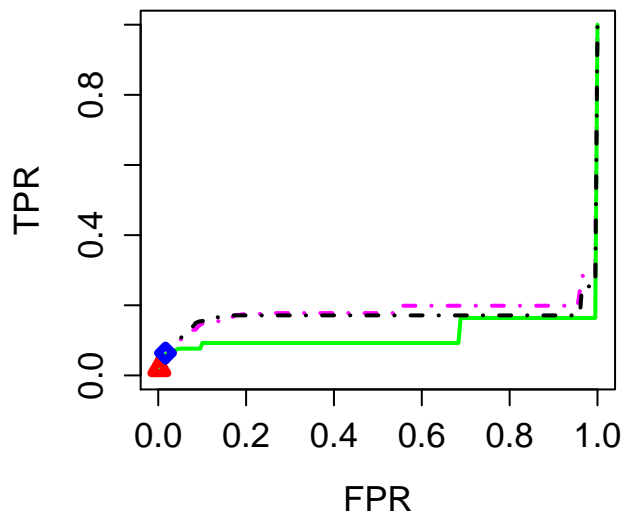

**n= 75**

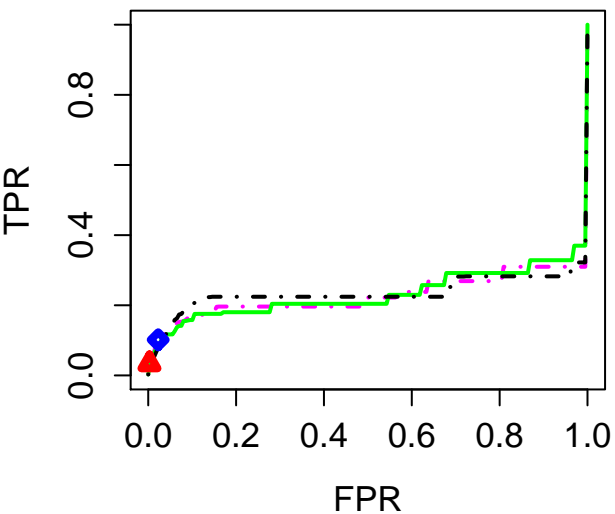

**n= 100**

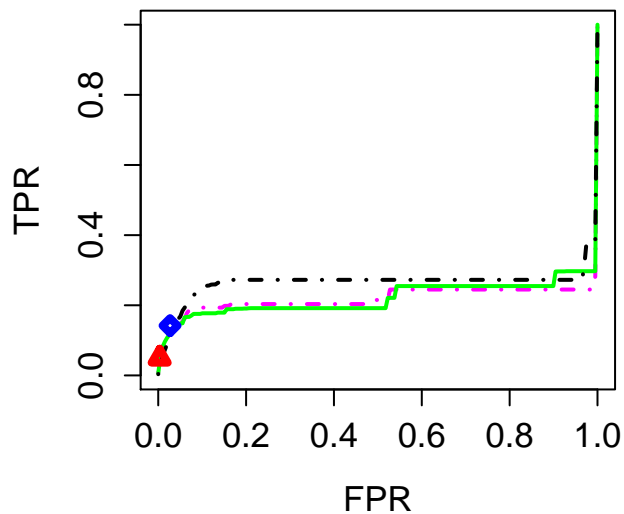

Supplement: Additional file 4 — ROC-curves for a density of 10%, part I. The figures display the ROC-curves for a density of 10%, and for n = 25; 50; 75; 100 [file 1471-2105-10-384-S4.PDF]

**n= 25**

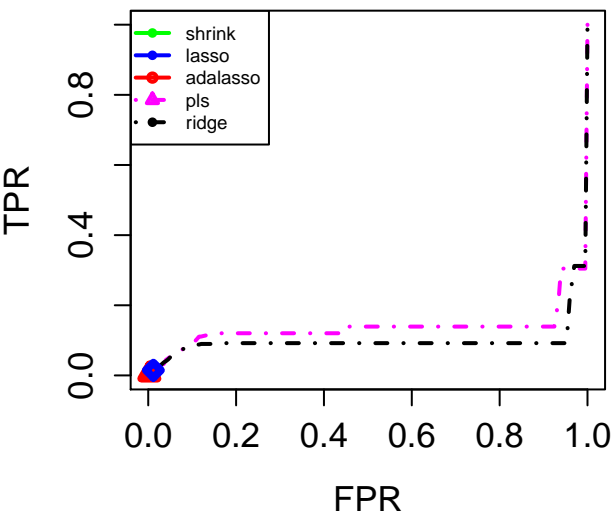

**n= 50**

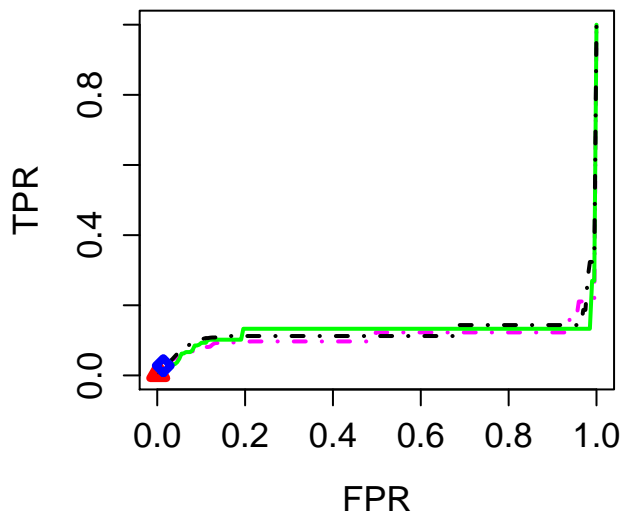

**n= 75**

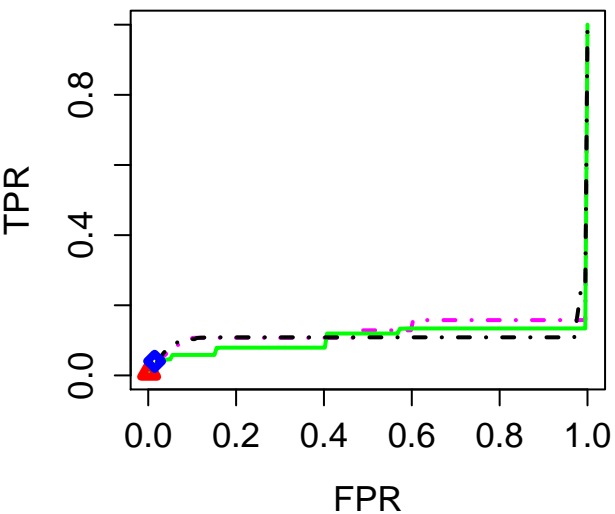

**n= 100**

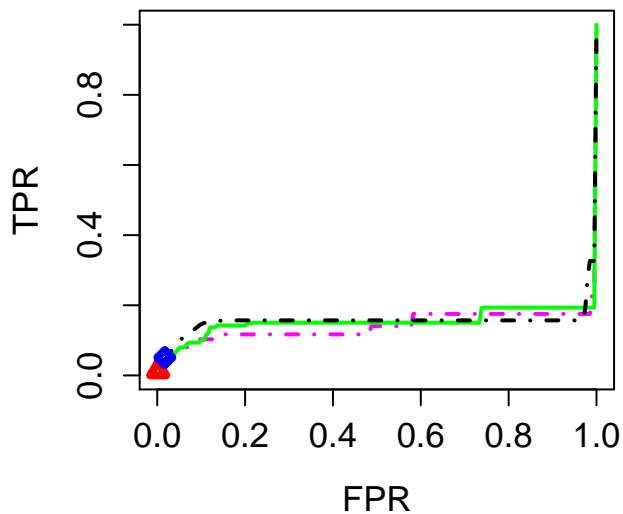

Supplement: Additional file 5 — ROC-curves for a density of 15%, part I. The figures display the ROC-curves for a density of 15%, and for n = 25; 50; 75; 100 [file 1471-2105-10-384-S5.PDF]

**n= 25**

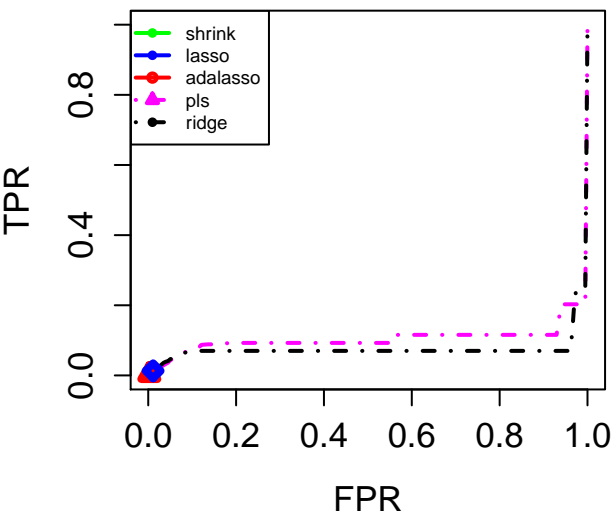

**n= 50**

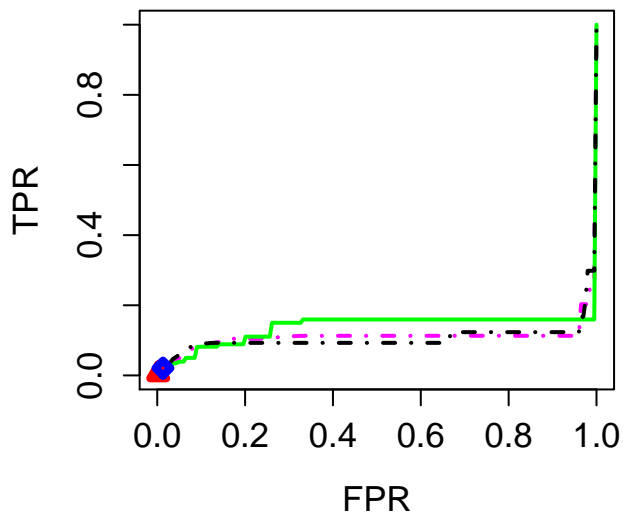

**n= 75**

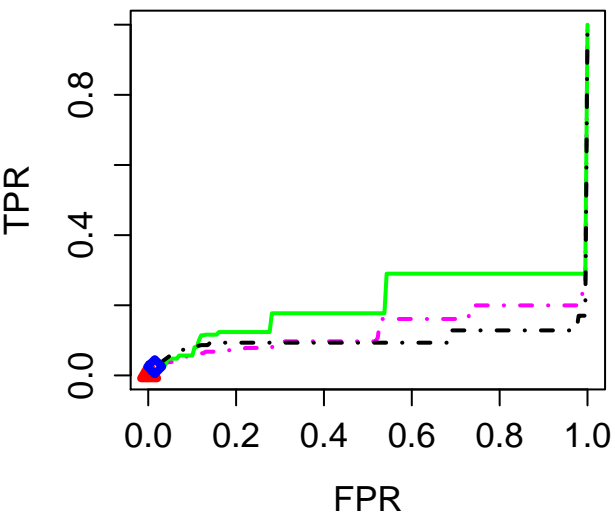

**n= 100**

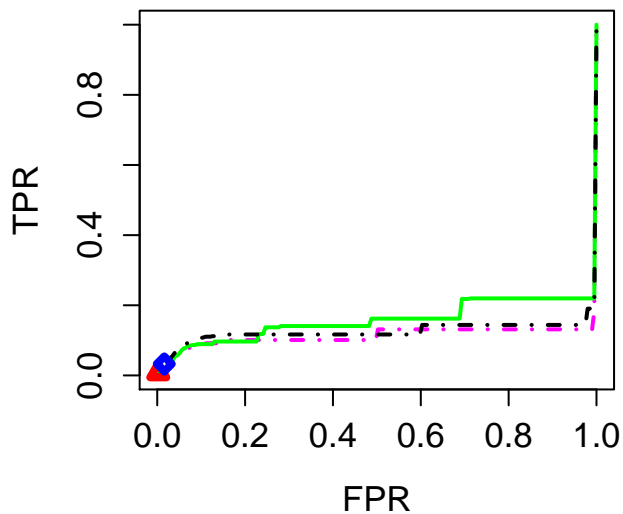

Supplement: Additional file 6 — ROC-curves for a density of 20%, part I. The figures display the ROC-curves for a density of 20%, and for n = 25; 50; 75; 100 [file 1471-2105-10-384-S6.PDF]

**n= 25**

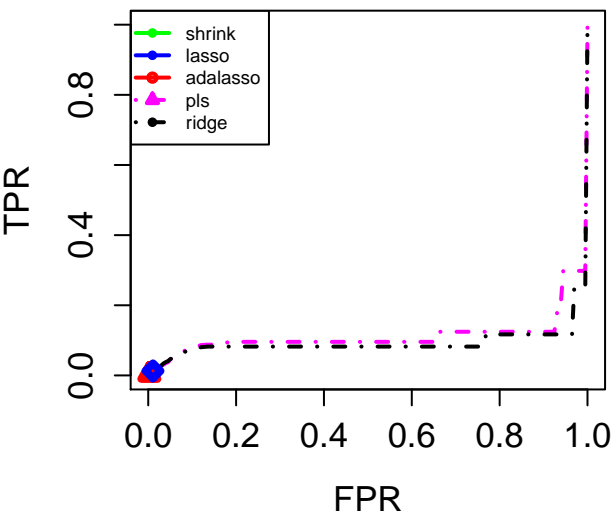

**n= 50**

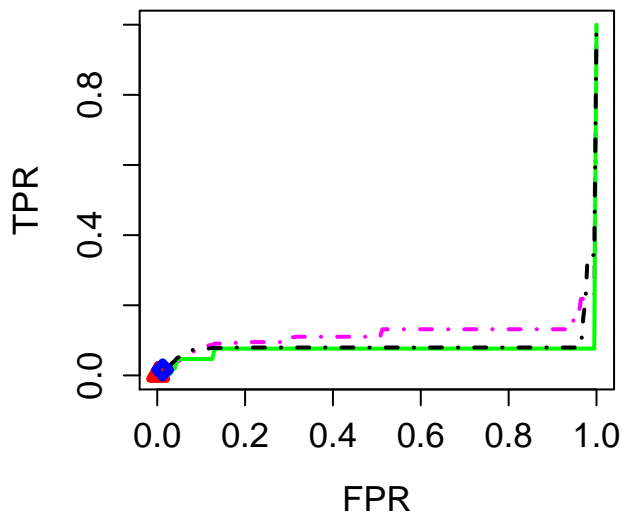

**n= 75**

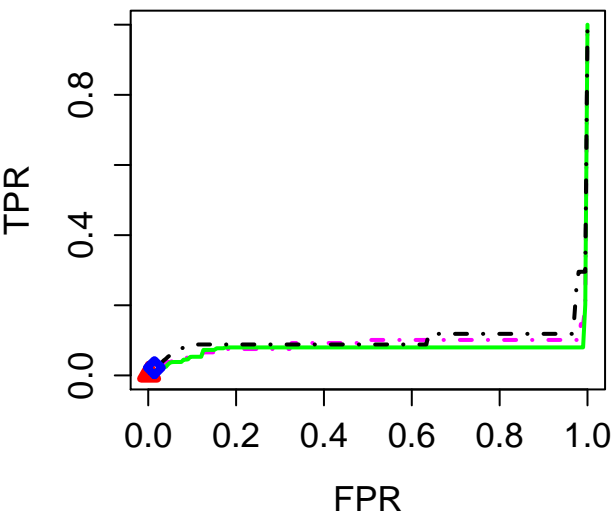

**n= 100**

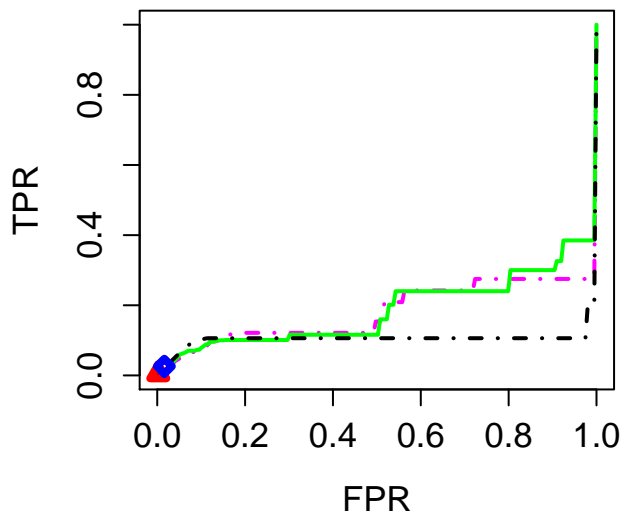

Supplement: Additional file 7 — ROC-curves for a density of 25%, part I. The figures display the ROC-curves for a density of 25%, and for n = 25; 50; 75; 100 [file 1471-2105-10-384-S7.PDF]

**n= 125**

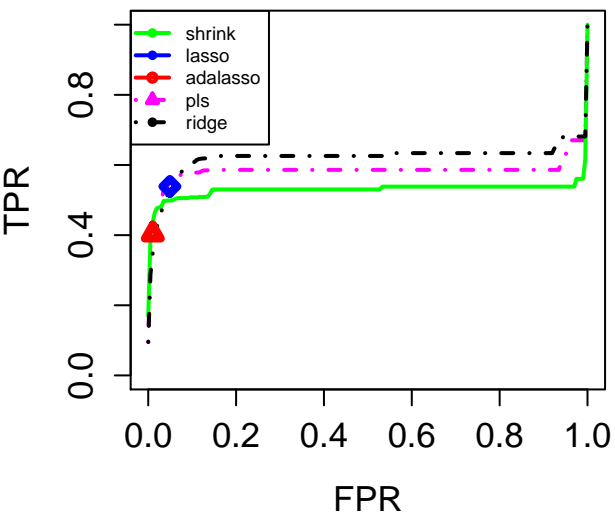

**n= 150**

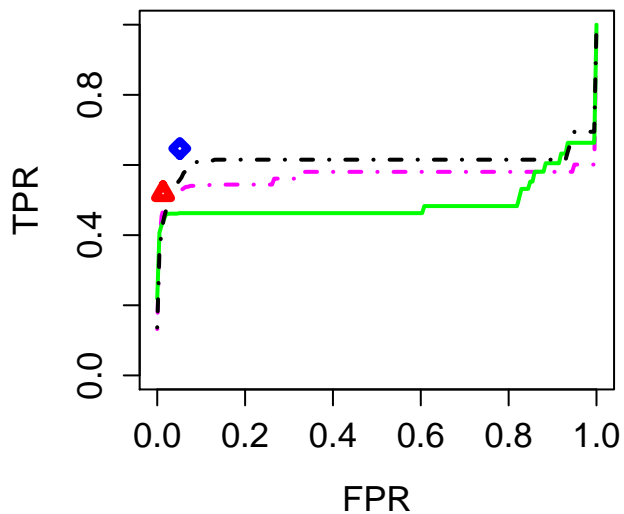

**n= 175**

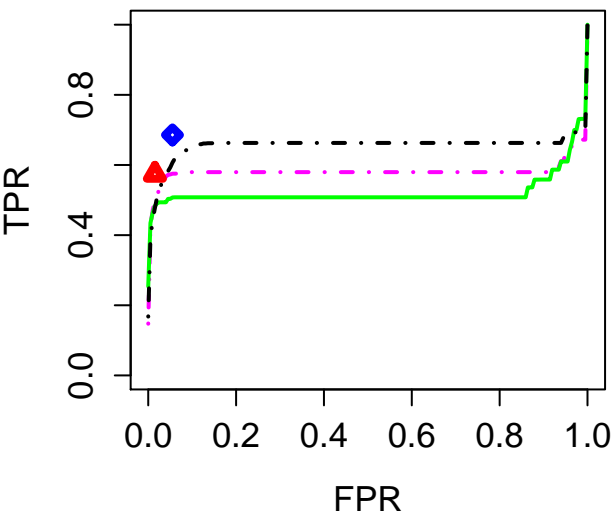

**n= 200**

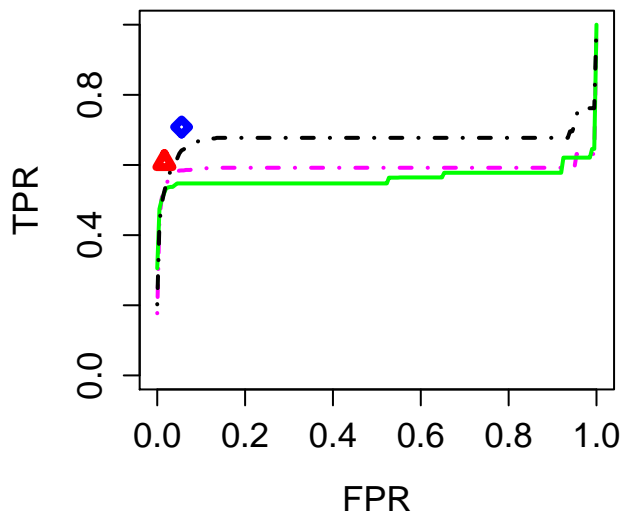

Supplement: Additional file 8 — ROC-curves for a density of 5%, part II. The figures display the ROC-curves for a density of 5%, and for n = 125; 150; 175; 200 [file 1471-2105-10-384-S8.PDF]

**n = 125**

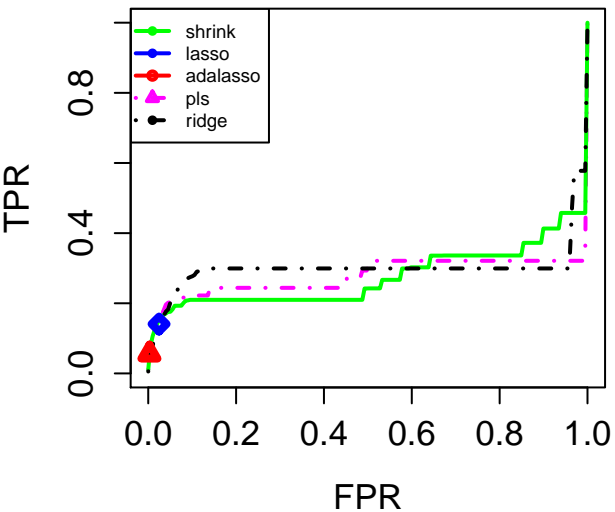

**n = 150**

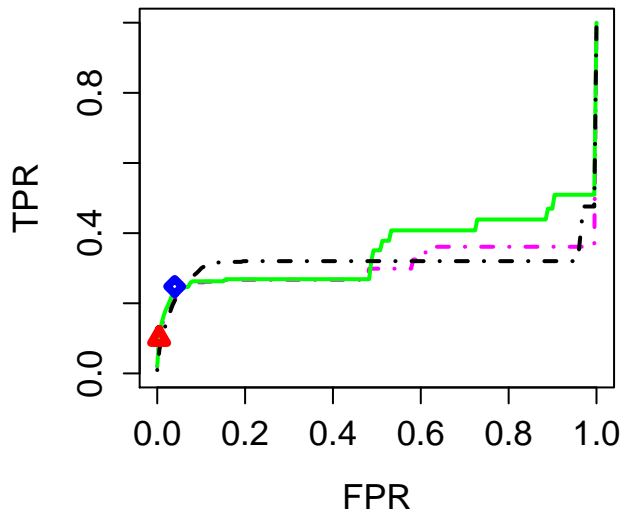

**n = 175**

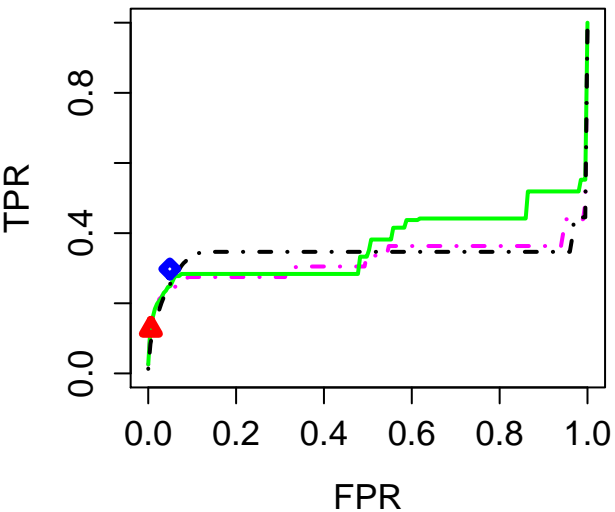

**n = 200**

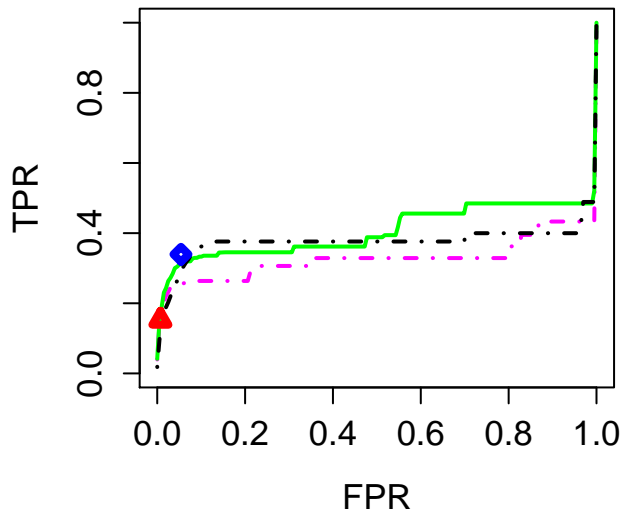

Supplement: Additional file 9 — ROC-curves for a density of 10%, part II. The figures display the ROC-curves for a density of 10%, and for n = 125; 150; 175; 200 [file 1471-2105-10-384-S9.PDF]

**n= 125**

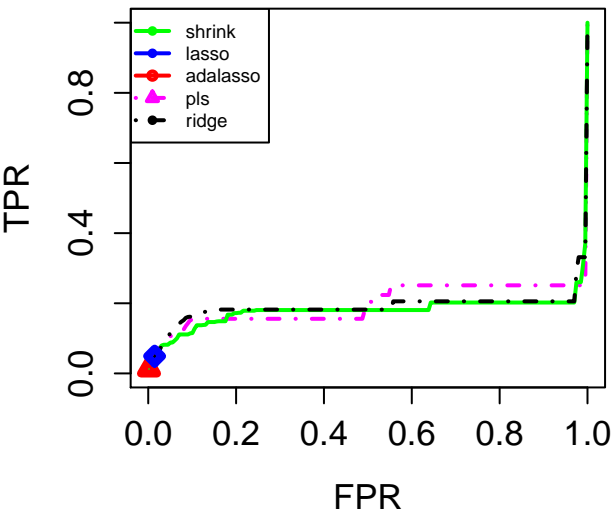

**n= 150**

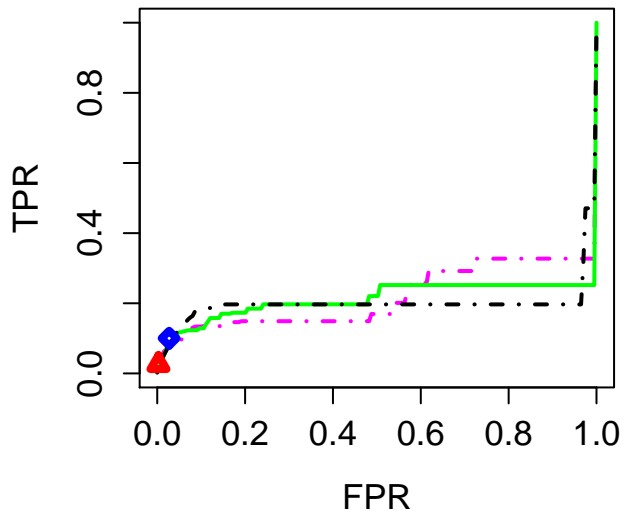

**n= 175**

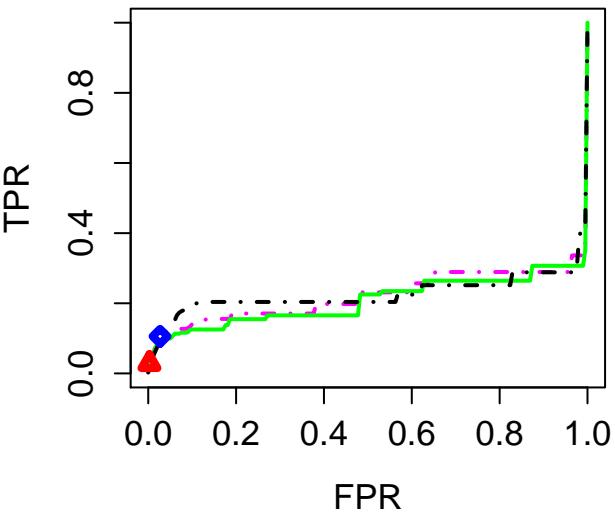

**n= 200**

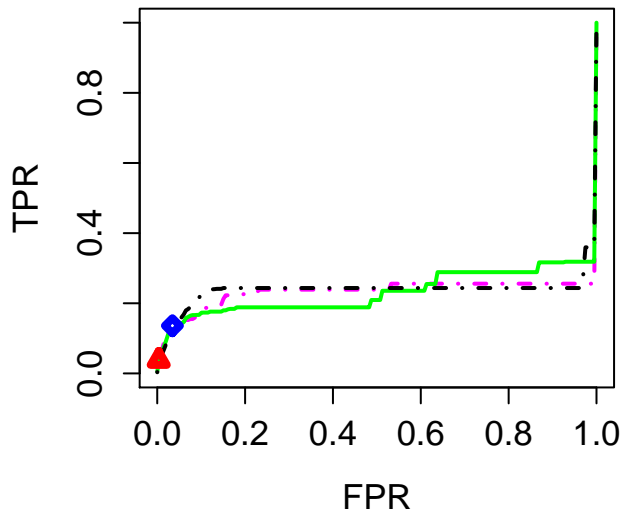

Supplement: Additional file 10 — ROC-curves for a density of 15%, part II. The figures display the ROC-curves for a density of 15%, and for n = 125; 150; 175; 200 [file 1471-2105-10-384-S10.PDF]

**n= 125**

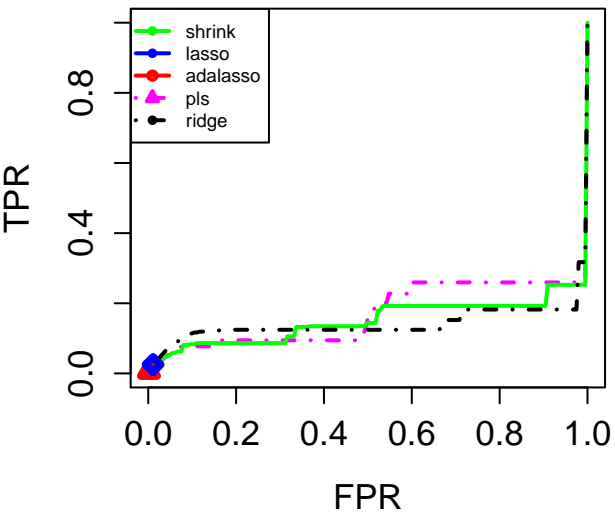

**n= 150**

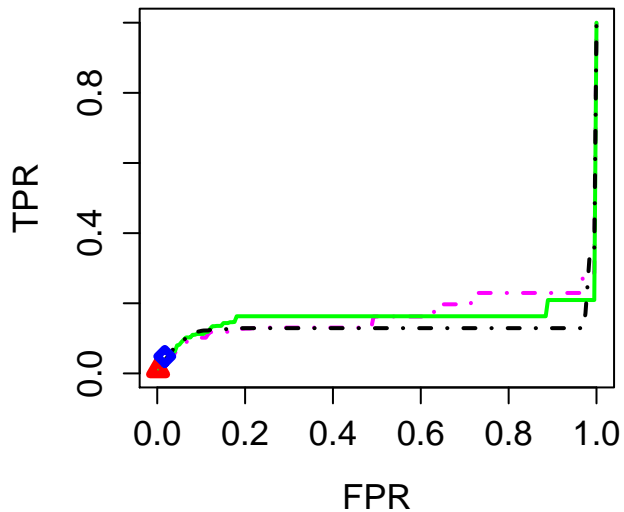

**n= 175**

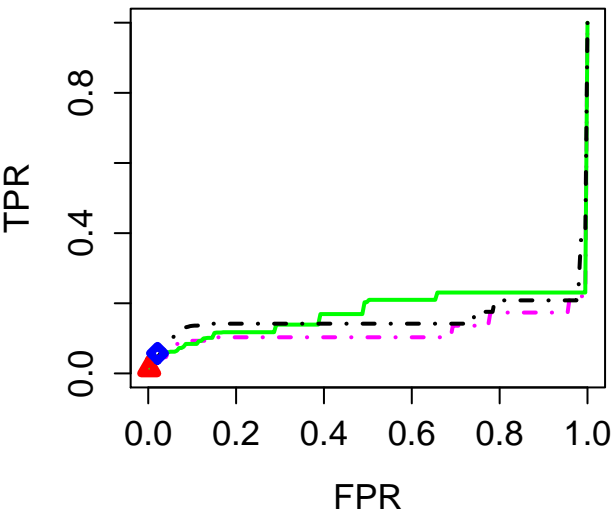

**n= 200**

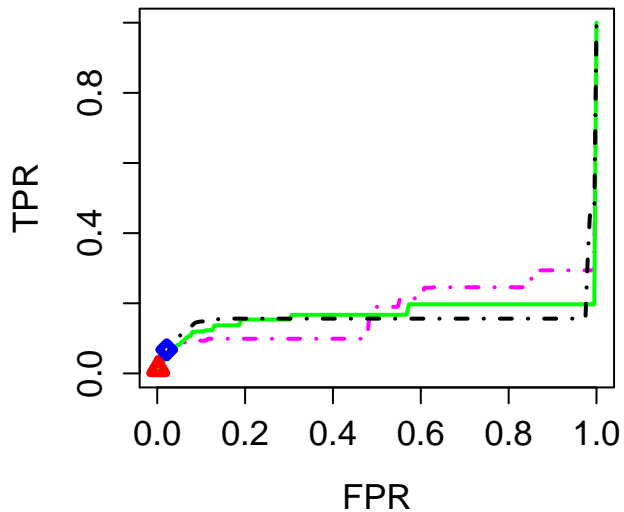

Supplement: Additional file 11 — ROC-curves for a density of 20%, part II. The figures display the ROC-curves for a density of 20%, and for n = 125; 150; 175; 200 [file 1471-2105-10-384-S11.PDF]

**n= 125**

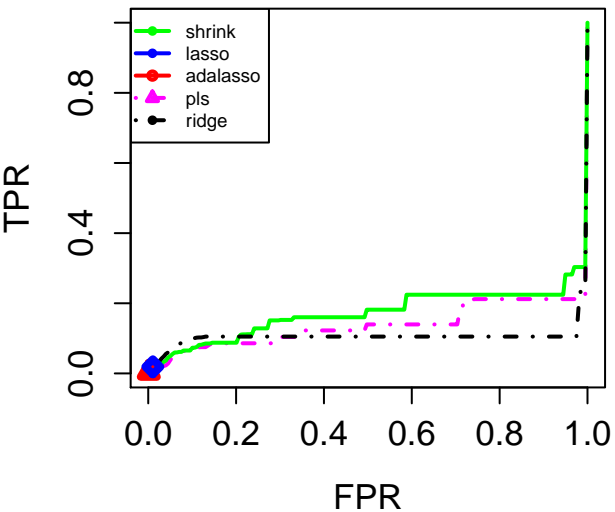

**n= 150**

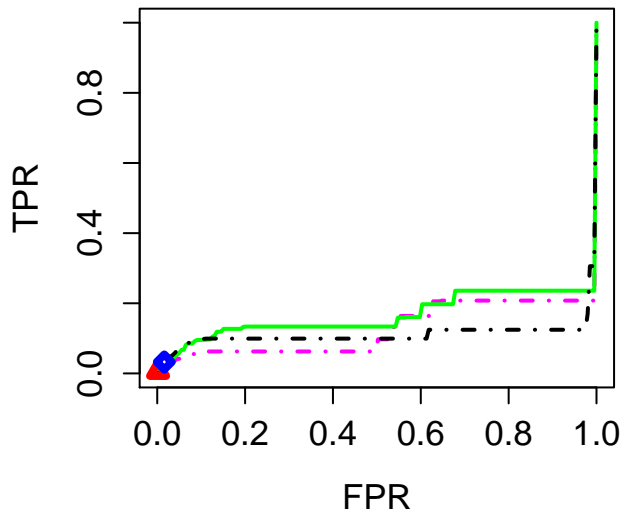

**n= 175**

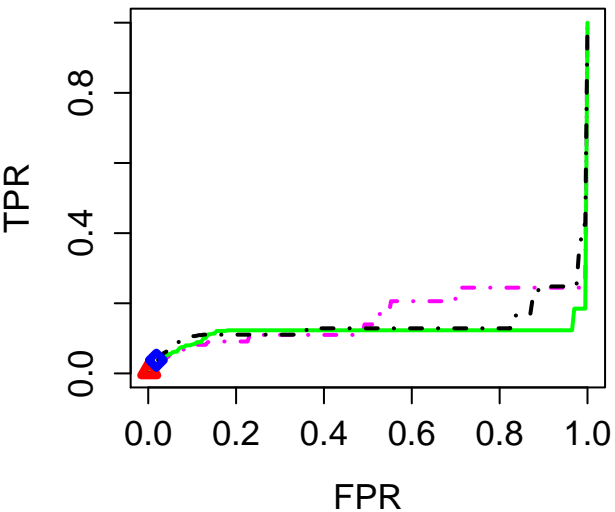

**n= 200**

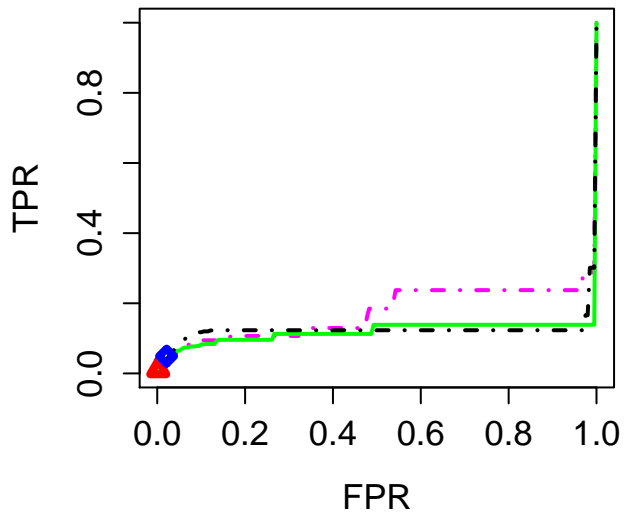

Supplement: Additional file 12 — ROC-curves for a density of 25%, part II. The figures display the ROC-curves for a density of 25%, and for n = 125; 150; 175; 200 [file 1471-2105-10-384-S12.PDF]
